# Rheumatic Bronchitis

**Published:** 1873-06-15

**Authors:** N. S. Davis

**Affiliations:** Professor of Clinical Medicine, Etc.


### Clinical Lectures.

### RHEUMATIC BRONCHITIS.

LECTURE DELIVERED IN MEDICAL WARDS OF MERCY HOSPITAL. BY N. S. DAVIS, M.D., PROFESSOR OF CLINICAL MEDICINE, ETC., MAY 30, 1873,

CASE I.—Mr. B——, aged about 35 years; native of Ireland; sanguine-lymphatic temperament; accustomed to the free use of fermented drinks; after an exposure to cold and wet was taken with rigors, followed by fever and pain in the left hypochondriac region, and was brought to the hospital one week since. The attention of the class was called to him the day after his admission. He then presented a deeply suffused redness of the face; a somewhat bloated aspect; temperature two or three degrees above the natural standard; pulse 105 per minute, and moderately full; respiration short, and a little accelerated in frequency; tongue covered with a white coat; bowels natural; urine scanty and high-colored; frequent but short and stifled cough, from a sharp and severe pain in the left side, directly in the line of the attachment of the diaphragm to the ribs. Percussion revealed no perceptible dulness over any part of the left side of the chest, and oscultation detected neither the friction of pleurisy nor the rales of pneumonia.

*Diognosis.*—The severe pain in the side, greatly aggravated by coughing, or attempting to take a full inspiration, with scanty expectoration, and some fever, suggested the existence of pleurisy; but as the patient had already been sick four or five days, which is sufficient to cause exultation either serous or plastic, from the surface of the inflamed membrane, and yet there being no physical signs of either this or of pneumonia, it was inferred that the patient was laboring under rheumatic fever, with local inflammation in the attachment of the left part of the diaphragm to the ribs. He was ordered a saline solution, alternately with one grain of calomel, with opium sufficient to lessen the severity of the pain in the side.

During the first two days the pain was diminished, and the patient appeared to progress favorably. After that, however, his breathing became more oppressed, with sharp sub-mucous rhonch over the whole of the left half of the chest; a quick pulse; short and hurried respiration; deep purple redness of the face; drowsiness, and slight delirium. The urine remained scanty and high-colored, and the patient was very restless. The cough was harsh and suffocating; the expectoration scanty and tenacious, and without any intermixture of blood. Neither was there any dullness or percussion over the affected side. The suddenness and severity of the dyspnœa; the scantiness of expectoration; the tight, suffocative cough, and the absence of dullness or percussion, led us to attribute the more prominent symptoms to an extension of the rheumatic inflammation to the bronchial tubes, including the smaller ramifications throughout the left lung. The patient was ordered the following prescriptions:

℞.—Liquor ammonii acetate, ℥ ii.

Tinct. opii et camph. ℥ iss.

Colchici. vin. ℥ ss.—Mix.

Give one fluid drachm every four hours,

℞.—Ammonia hydrochl. ℥ iii.

Ant. tart. et pot, grs. ii.

Morph. sulph. grs. iii.

Glycyrrhiza syr. ℥ iv.—Mix.

Give one fluid drachm every four hours, alternating with the other prescription.

In consequence of the coolness of the extremities, and the sweating, he was also given three grains of quinine three times a day, and a blister was applied to the lower part of the left side of the chest. He has continued this treatment for the last four days, with a steady improvement in all the symptoms.

Now, we find the temperature of the body and extremities nearly natural; the flush nearly gone from the face; the pulse 98, and soft; the pain in the side only slight; but the breathing still oppressed; the cough harsh, but less frequent; and the patient complaining of great weakness. Percussion still shows a fine degree of resonance, while auscultation gives a well-marked sub-mucous rhonchus over all the middle and lower parts of the left side, with piping sounds, mixed with a coarser mucous rhonchus over the upper and anterior part, The same treatment was continued, except the interval between the doses of the liquid medicines was increased to six hours, and the quinine reduced to two grains instead of three.

At a subsequent clinic, one week later, this patient was found fully convalescent.

CASE II.—Mr. C——, a laborer; native of Ireland; aged 45 years; was admitted into the hospital yesterday; reported to have pleurisy. By simple inspection the patient was found to occupy a dorsal position, with the head and shoulders a little elevated; the face and lips pale; the whole body thin in flesh; the expression of countenance dejected and sad; the mouth and tongue moist; the respiration labored, the inspiratory act being forced with depression of the intercostal and supra-clavicular spaces, at its beginning, and the expiratory act being prolonged, and wheezing, as in asthma; and the left side of the chest was seen to expand much more than the right. By touch, the temperperature was found nearly natural, the extremities rather cool, and the pulse 110 per minute, small and weak. By percussion, the resonance was found natural on both sides of the chest; but auscultation revealed exaggerated respiratory murmur, with some dry rhonchi in the left side, while all respiratory sounds were absent from the lower, lateral and posterior parts of the right side, and a feeble murmur, mixed with some dry sounds, over the anterior and upper part of the same side. The patient had been attacked about one week previously with a chill, followed by some fever and severe pain through the central part of the right half of the chest, which still continues, and is accompanied by a disturbing cough and sense of exhaustion. His bowels were inactive, and the urine less than natural.

It was remarked that the combination of symptoms in this case was unusual. The sudden attack of severe pain in the right chest, five or six days since, with the present absence of all respiratory sounds over the middle and lower parts of that side, would certainly suggest the existence of pleuritic inflammation, accompanied by effusion. But serous effusion, sufficient to compress the lung and suppress the respiratory murmur, would necessarily be accompanied by dullness on percussion, fullness of the side, and shortness of both inspiration and expiration; while the reverse of all these conditions exist in this case. That the absence of respiratory sounds does not depend on complete hepatization is evident, for this would also involve dullness on percussion, as well as bloody expectoration and shortness of expiration. The prolonged expiratory act, the sinking of the intercostal spaces, and the imperfect expansion during inspiration, with the resonance, would suggest the existence of some cause affecting the right side of the chest in such a manner as to shut off the air from reaching the air-cells in the middle and lower part of the right lung. Such a result might be produced by aneurismal or other tumors pressing on the larger bronchial tubes; by inflammation, either rheumatic or diphtheritic, causing a fibrinous exudation upon the bronchial membrane sufficient to cause exclusion of air; or, possibly, by embolus in the right division of the pulmonary artery, preventing the supply of blood to the capillary net-work surrounding the cells. The existence of aneurismal or other tumors would be readily detected by auscultation and percussion, and hence must be excluded from this case. From all the facts, as we get them in the history and present condition of this case, we are inclined to regard it as one of rheumatic inflammation of the right bronchial tubes, accompanied by so much fibrinous or plastic exudation as to nearly exclude the air from the affected part of that side.

If so, the prognosis is very unfavorable, because the plastic and tenacious character of the exudation, in accute rheumatism, make it slow and difficult of disintegration and absorption; and a very moderate extension of the present obstruction to the respiratory functions in this case would prove fatal. The patient was ordered a mixture of liquor ammonia ascetatis two parts, and camphorated tincture of opium one part, a fluid drachm every two hours, and two grains of quinine three times a day, with moderate counter-irritation over the right side. The treatment recommended appeared to have no perceptible effect. The difficulty of breathing, and prostration, continued to increase, and the case terminated fatally two days after the clinic. No *post-mortem* was allowed.

